# HPV, APOBEC3B, and the origins of breast cancer: a narrative review and perspectives on novel mechanisms

**DOI:** 10.3389/fonc.2025.1737189

**Published:** 2026-01-12

**Authors:** Zhi-yong Liu, Ran Chen

**Affiliations:** Department of Breast Treatment, The First Affiliated Hospital of Gannan Medical University, Ganzhou, Jiangxi, China

**Keywords:** APOBEC3B, breast cancer, human papillomavirus, prevention, review, viral carcinogenesis

## Abstract

Breast cancer is one of the most severe global health challenges, with its incidence continuing to rise. The development of this malignancy is associated with numerous risk factors; however, its primary etiology often remains unclear. Research on the potential association between breast cancer and carcinogenic human papillomavirus (HPV) has been conducted for three decades, yet a definitive consensus has not been reached. The development of prophylactic cervical cancer vaccines has greatly heightened the urgency of this issue: if a causal relationship can be established, it would open a feasible avenue for preventing this common cancer. This review aims to systematically integrate relevant evidence and provide a critical commentary on the association between HPV and breast cancer. We analyzed evidence from 34 studies on HPV DNA detection in breast cancer cells. We discuss the prevalence and genotypes of HPV detected in breast cancer across different geographical regions, the state of the viral genome (integrated vs. episomal), viral load, and potential mechanisms for HPV-associated breast carcinogenesis—particularly its role in inducing genomic instability via activation of APOBEC3B. Additionally, we explore the histopathological and clinical characteristics of HPV-positive breast cancer. The synthesized evidence suggests that high-risk HPV types are present in a subset of breast cancers worldwide, but with lower viral loads compared to cervical cancer, indicating a potentially different mode of action. The association between HPV and breast cancer warrants further rigorous investigation to clarify its clinical and preventive implications.

## Introduction

As the most common malignant tumor among women globally, the incidence of breast cancer shows an annual increasing trend (1). Currently, treatment methods for breast cancer are continuously innovating, with significant progress in surgical treatment, chemotherapy, endocrine therapy, targeted therapy, and immunotherapy, greatly improving the prognosis of breast cancer patients (2). However, some patients still develop drug resistance during treatment and have poor outcomes (3). Breast cancer is a highly heterogeneous disease characterized by dysregulation of numerous genes and expression cascades (4, 5). Therefore, there is an urgent need to explore factors closely related to the occurrence, development, and prognosis of breast cancer and to elucidate their underlying mechanisms in depth.

The concept of viral involvement in the development of such tumors dates back to the research of American investigator J. Bittner, who identified the causative agent (Bittner virus, also known as the “milk factor”) of mouse mammary cancer. In this model, the importance of various factors in tumor development was elucidated, including viral factors, the specific genotype of the mouse, its inbred strain, and the hormonal background of the animal (6, 7). Subsequently, viruses similar or related to the mouse mammary tumor virus were detected in humans (8). Existing data suggest an association between breast cancer and Epstein-Barr virus (9, 10).

In 1996, the World Health Organization clearly identified high-risk human papillomavirus (HPV) as a carcinogen and the cause of cervical cancer. This conclusion was based on the detection of HPV DNA in the majority of cervical cancer samples studied (11). Since then, the list of known cancer-causing HPV types in humans has been continuously updated, as has the list of cancer types caused by them (12–14). HPV types 16 and 18 are detected in about 70% of cervical cancer samples; HPV types 31, 33, 35, 39, 45, 51, 52, 56, 58, and 59 are also recognized as human carcinogens; for several other HPV types such as 26, 53, 66, 67, 68, 70, 73, and 82, evidence for carcinogenicity is limited; HPV types 6 and 11 are classified as low-risk carcinogenic viruses (14). Breast cancer is classified as a tumor type with insufficient evidence, currently insufficient to conclude that it is “associated with papillomaviruses” (12).

Besides HPV, other viruses have been studied for a possible association with breast cancer. For example, Epstein-Barr virus (EBV) has been detected in some breast cancer samples, but its pathogenic role and mechanisms are still under investigation (9, 10). In recent years, the role of human cytomegalovirus (HCMV) in tumorigenesis has also garnered attention. HCMV is a herpesvirus that, in addition to causing disease in immunocompromised patients, encodes proteins (such as IE1, IE2, US28) with oncogenic properties capable of activating pro-oncogenic pathways like STAT3 and PI3K/Akt, inducing cell proliferation, inhibiting apoptosis, promoting angiogenesis and immune evasion, fulfilling multiple hallmarks of cancer (15). Although direct evidence linking HCMV to breast cancer is less substantial than for HPV, studies suggest that HCMV infection may influence the breast cancer microenvironment or process through mechanisms of “oncomodulation” or direct transformation (15). Exploring multiple viral factors contributes to a more comprehensive understanding of the complex etiology of breast cancer.

Human papillomavirus is a species-specific, epithelium-tropic virus belonging to the family Papillomaviridae. The viral particle has a diameter of 50–60 nm, with its DNA enclosed within a protein capsid (16). Classification of HPV is based on the degree of nucleotide sequence mismatch in the L1 gene, the most conserved region of the genome. Researchers pay particular attention to over 120 HPV types (17). HPV can be divided into five genera. Alpha-papillomaviruses primarily infect oral and anogenital mucosa; all known carcinogenic HPV types belong to the alpha genus. The HPV genome is a circular double-stranded DNA molecule approximately 8 kilobase pairs in size, consisting of three regions: 1) the long control region, containing sequences regulating viral replication and transcription; 2) the early (E) region, containing open reading frames for the E1, E2, E4, E5, E6, and E7 genes, which play key roles in the “early” functions of the virus, including viral genome replication and transcription, as well as conferring malignant characteristics to cells infected with carcinogenic HPV; 3) the late (L) region, encoding the structural proteins L1 and L2 of the viral capsid. The primary route of HPV infection is direct contact of damaged (fissured or wounded) epithelium with HPV-infected epithelial cells (18). Transformation of infected cells requires long-term HPV infection and sustained expression of the viral oncoproteins E6 and E7. The activities of the E6 and E7 protein products vary among HPV types; these evolutionary differences lead to the classification of HPV into “low” and “high” carcinogenic risk types. For carcinogenic HPV, the indicator of “viral load” (the number of viral genomes per infected cell) has been established to be positively correlated with the risk of progression of virus-induced lesions to cancer. The oncoproteins E6 and E7 of high-risk HPV have short half-lives and can bind to and interfere with the functions of multiple host cell proteins (19). The oncoprotein E7 interacts with the tumor growth suppressor protein retinoblastoma protein (pRB), which regulates the activity of the E2F family of transcription factors. Binding of E7 to pRB promotes its degradation, allowing the cell to pass smoothly through the G1/S checkpoint of the cell cycle (20). The oncoprotein E6 interacts with the tumor growth suppressor protein p53, leading to its rapid degradation (21). E6 can activate the transcription of the telomerase catalytic subunit hTERT, enabling cells to rebuild telomere regions of chromosomes and acquire “immortal” characteristics (19). Both E6 and E7 oncoproteins significantly enhance genomic instability in host cells (19, 22). This can be achieved in two ways. First, in p53-deficient cells, genomic repair processes are impaired, leading to increased frequency of gene mutations. Second, the E7 oncoprotein can interfere with centriole replication in infected epithelial cells, resulting in multipolar mitoses and aneuploidy.

A. Di Lonardo and colleagues published the first report on the potential association between human papillomavirus and breast cancer in 1992 (23). Since then, researchers from various countries have obtained data on HPV positivity in breast cancer. Following the advent of vaccines effective for cervical cancer prevention, the significance of clarifying the role of high-risk HPV in breast cancer pathogenesis has increased further, given its potential for preventing this common cancer (24). This review aims to analyze the current status and future directions of research on the association between HPV and breast cancer, and to integrate relevant evidence for commentary.

## Methods

This article aims to systematically organize and comment on existing research evidence regarding the association between HPV and breast cancer, rather than being a systematic review or meta-analysis following a strictly predefined protocol. Literature searches were conducted in the PubMed, Web of Science, and Google Scholar databases using the keyword combinations: “human papillomavirus” OR “HPV” AND “breast cancer” OR “breast carcinoma”, with a time frame up to 2024. Inclusion criteria were: original studies (case-control, cross-sectional, etc.) investigating the detection of HPV DNA or protein in human breast cancer tissues, cell lines, or related samples; published in English; providing clear HPV detection methods and positivity rate data. Exclusion criteria were: non-original articles (e.g., reviews, editorials), conference abstracts, non-human studies, studies not providing specific detection data or with unclear method descriptions.

Initial screening was performed by reading titles and abstracts, and eligible articles were further obtained in full text for assessment. A total of 34 studies were finally included. Data extraction included: first author, publication year, study country, sample type, sample size, HPV detection method, HPV positivity rate, main HPV genotypes, and key findings.

Due to high heterogeneity among the included studies in design (e.g., sample source, control settings), detection methods (type of PCR, primers, sequencing techniques), and populations (region, ethnicity), conducting a quantitative meta-analysis to derive a single pooled effect estimate could be misleading. Therefore, this article employs a descriptive synthesis approach, presenting data through tables and textual summaries, with particular attention to factors that may explain heterogeneity (such as geographic region, sensitivity of detection techniques). Simultaneously, this article acknowledges and discusses the phenomenon of vastly differing positivity rates among studies (ranging from 0% to over 86%), pointing out that methodological differences (especially detection sensitivity, primer coverage) are likely the primary reasons, rather than simply geographic or population distribution disparities. Although standardized tools were not used to assess the risk of bias for each study, the inherent limitations of observational studies, such as selection bias, misclassification bias due to detection method differences, and the inability of association studies to directly prove causation, are explicitly noted in the discussion.

## Detection of human papillomavirus DNA in breast cancer

Based on the methods described above, we included 34 studies. As shown in **Table 1**, the detection rate of HPV DNA in breast cancer tissues varied enormously among studies, ranging from 0% (25–27) to over 50% and higher (28–35). This variation makes direct comparison difficult and may be influenced by factors such as: sample size; whether the breast tissue was obtained fresh/frozen from biopsy or surgery, or was formalin-fixed and paraffin-embedded; and the characteristics of the HPV detection method, particularly its sensitivity and the range of HPV types it can detect. All studies, including those that did not detect HPV-positive breast tissue, controlled for the quality of extracted cellular DNA by using PCR with primers for the β-globin gene (25, 27) or spectrophotometry (26); samples with inadequate DNA quality were excluded.

**Table 1:** Data of human papillomaviruses DNA detection in breast cancer samples.

Country	Number of samples,n	Methods of detection	HPV+samples,%	Predominant HPVtype	Reference
Italy	17	PCR	29.4	HPV-16	(22)
Great Britain	80	PCR	0	–	(25)
India	228	PCR,real-time PCR	0	Only HPV-16 and HPV-18 were tested	(26)
Iran	150	PCR	0	–	(27)
Austria	11	PCR	63.6	HPV-16	(28)
Germany	29	PCR,*in situ* hybridization	86.2	HPV-11(HPV-16 and HPV-66 in rare cases)	(29)
Turkey	50	PCR	74.0	HPV-33	(30)
Hungary	1	Nested PCR,*in situ* hybridization	One HPV+ case	HPV-18,HPV-33	(31)
Syria	113	PCR	61.1	HPV-33	(32)
Spain	251	PCR,microchip typing	51.8	HPV-16	(33)
Australia	28	Nested PCR,*in situ* PCR, real-time PCR, sequencing	78.6	HPV-18	(34)
India	272	PCR,Southem blotting, multiplex PCR	63.9	HPV-16,HPV-18,and HPV-33 in descending order	(35)
Iran	72	PCR,typing with a commercial set	48.6	HPV-18,HPV-16,and HPV-33 in descending order	(38)
Iran	59	Nested PCR,real-time PCR, sequencing	11.8	HPV-18	(39)
Norway	41	PCR,*in situ* hybridization	46.0	HPV-16	(41)
China	32	PCR,Southern blotting	43.8	HPV-33	(42)
USA	101	PCR,sequencing	24.8	HPV-16	(43)
Greece	107	PCR,restriction fragmentlength polymorphism	15.9	HPV-16	(44)
Japan	124	PCR,real-time PCR,sequencing	21.0	HPV-16	(45)
Australia	28	PCR,*in situ* PCR,sequencing	28.6	HPV-18	(46)
Mexico	67	PCR,restriction fragmentlength polymorphism,sequencing	4.4	HPV-16,HPV-18,HPV-33	(47)
Mexico	20	PCR,real-time PCR	40	HPV-16	(48)
Venezuela	24	PCR	41.7	HPV-51	(49)
Australia	80	Nested PCR,sequencng	16	HPV-18	(50)
Korea	106	Real-time PCR	17.9	HPV-51	(51)
China	81	Hybrid Capture 2(HC2)	17.3	13 types ofoncogenic HPV in total	(52)
Thailand	350	PCR,typing with ELISA	4.3	HPV-16	(53)
Denmark	193	PCR with commercial kits	15.5	HPV-16	(54)
Marocco	76	Multiplex PCR	25.0	HPV-11;HPV-51, HPV-58, and HPV-59 one case each	(55)
Brazil	103	PCR,sequencing	49.5	HPV-6,HPV-11;rarely HPV-18 and HPV-33	(56)
Italy	273	*In situ* hybridization	44.4	HPV-16	(57)
Qatar	50	Multiplex PCR	10.0	HPV-16and HPV-35	(58)
Poland	383	Nested PCR,typing with a commercial set	4.4	HPV-16	(59)
Australia	855	Next generation sequencing, PCR, *in situ* PCR,real-time PCR	5.9	HPV-18	(60)

PCR, polymerase chain reaction; ELISA, enzyme-linked immunosorbent assay; HPV, human papillomavirus.

In breast cancer tissue samples, detection of human papillomavirus is typically performed using polymerase chain reaction (PCR) techniques. This technique often uses universal primer pairs targeting conserved regions of the HPV L1 open reading frame, such as GP5/6 or MY09/11 primer pairs, to detect various HPV types (14). In such cases, these primer pairs can be used for nested PCR. Additionally, type-specific primers targeting regions like E6 and E7 are also frequently employed.

A. Di Lonardo and other researchers, using PCR technology, detected human papillomavirus DNA in 29.4% of breast cancer samples; HPV was also detected in the axillary lymph nodes of some patients, indicating the onset of metastasis. However, when the authors attempted to verify this result using *in situ* hybridization, HPV DNA was not detected in any sample (23). This study underscores the importance of selecting an appropriate HPV detection method.

The sensitivity of all human papillomavirus DNA detection methods and the direct laboratory experience of researchers appear to be among the primary reasons for the significant differences in the frequency of HPV-positive breast cancer samples reported by different authors. Developing multiple HPV detection methods to achieve comparable results across laboratories and improving these methods remains an important task (36, 37). Significant fluctuations in the frequency of HPV-positive breast cancer cases are observed even within studies from the same country [e.g. (26, 27, 35, 38, 39)], indirectly suggesting that the cause is not racial and geographic distribution disparities of breast cancer, but rather differences in detection methodologies.

Collaborative advancements in human papillomavirus genomics and epidemiology will inevitably impact research on the HPV-breast cancer association. Simultaneously, the academic understanding of HPV-induced carcinogenesis mechanisms has deepened, shifting from a view of “evolutionary stasis” to recognizing “the existence of thousands of unique viral genomes with varying oncogenic potential.” For instance, in the most widely studied HPV16 (accounting for no less than 50% of global cervical cancer cases), currently known nucleotide diversity variations have been classified into 4 major lineages and 16 sub-lineages, each with distinct virus-host interaction, tissue tropism, splicing, and translation process characteristics (40). When improving detection methods for such viruses, the latest nucleotide diversity data of HPV will be considered. Related studies have confirmed that breast cancer samples can be co-infected with multiple different HPV types (**Table 2**).

**Table 2:** Breast cancer coifection with several human papillomaviruses (HPV) types.

Country	Number of samples with several HPV types/all HPV+samples	Predominant HPV typesincoinfection	Reference
Germany	8/25	HPV-6,-11,-16	(29)
Syria	24/69	HPV-16,-18,-31,-33,-35	(32)
Spain	85/130	HPV-16,-31,-39,-51,-59	(33)
India	44/174	HPV-16,-18,-33	(35)
USA	1/25	HPV-16,HPV-18	(43)
Greece	3/17	HPV-16,-58,-59,-73,-82	(44)
Kopes Korea	10/22	HPV-6,-33,-51,-53,-58	(51)
Thailand	4/15	HPV-16,-35,-18,-33,-52	(53)
Morocco	8/19	HPV-6,-11,-52,-58,-59	(55)
Karap Qatar	2/5	HPV-16,-35,-58	(58)

Multiple research teams have detected viral DNA in non-randomly selected breast tissue samples. This review focuses only on the following scenario: during initial histological examination of patients, koilocytes (flat epithelial cells) were found in both normal and cancerous breast cells. These cells undergo a series of morphological changes in the nucleus and cytoplasm due to infection by specific types of human papillomavirus, which is an accepted cytological feature of cervical epithelial HPV infection (61). E.M. de Villiers and her team (29) conducted research using this approach.

In such samples, human papillomavirus positive results are extremely common in breast cancer samples: 86.2% (29) (predominantly low-risk HPV), 46.0% (41), 78.6% (34), and 63.6% (28), respectively. This suggests that HPV types found in breast cancer align with those found in cervical dysplasia and cancerous epithelium, supporting non-traditional hypotheses about HPV transmission in women (28, 31, 34, 41).

For invasive breast cancer, besides detecting the primary tumor lesion, attempts have been made to detect human papillomavirus DNA in regional lymph nodes and/or distant organ metastases of breast cancer (23, 28, 41). As mentioned earlier, A. Di Lonardo and colleagues detected HPV16 DNA in the axillary lymph nodes of 2 out of 5 invasive breast cancer patients, both of whom carried the virus (23). E.M. Hennig and colleagues detected HPV16 in regional metastases of 4 out of 7 HPV-positive primary breast cancer patients; HPV16 was also detected in a distant metastasis (located in the colon) of one patient; in 13 patients whose primary tumors were HPV-negative, no virus was detected in their metastases (41). A. Widschwendter and colleagues detected HPV16 DNA in the axillary lymph nodes of 2 breast cancer patients, and HPV16 was also detected in the primary tumor lesions of these 2 patients (28).

Multiple research groups have not only tested breast cancer samples for human papillomavirus but have also used normal breast tissue obtained from reduction mammoplasty in healthy women as a control group for detection (**Table 3**). In four out of five relevant studies, DNA of carcinogenic HPV types (types 18 and 35) was detected in normal breast tissue. In all these studies, the detection rate of HPV-positive samples in normal breast tissue was lower than the rate of HPV-positive breast cancer cases (24.8%, 28.6%, 48.6%, 49.5%, and 10.0%, respectively) (see **Table 1**). Simultaneously, the predominant HPV types matched those detected in cancerous tissue samples. As reference groups, some researchers also used conditionally normal breast tissue adjacent to breast cancer (**Table 4**), or breast tissue surgically removed for non-malignant diseases (such as breast hyperplasia, fibroadenoma, and lipoma) (**Table 5**). The detection rate of HPV in conditionally normal breast tissue adjacent to cancer and in normal breast epithelium from healthy women is not low (see **Table 4**). However, in all studies, the HPV detection rate in these samples was lower than in the adjacent breast cancer tissue (86.2%, 74.0%, and 63.9%, respectively) (see **Table 1**). The HPV types found in these tissues typically did not differ from those found by the same authors in breast cancer tissues.

**Table 3:** Data of human papillomaviruses (HPV) DNA detection in normal mammary gland tissue of healthy women.

Country	Number of samples, n	HPV+samples,%	Predominant HPVtype	Reference
Iran	31	16.1	HPV-18	(38)
USA	20	0	–	(43)
Australia	17	18.0	HPV-18	(46)
Brazil*	95	15.8	HPV-6,-11,-18	(56)
Qatar	50	8.0	HPV-35	(58)

It was not possible to determine the type of HPV in every sample.

**Table 4:** Data of human papillomavirus (HPV) DNA detection in conventionally normal tissue of breast cancer patients.

Country	Number of samples, n	HPV+samples,%	Predominant HPV type	Reference
Germany	29*	70.0	HPV-11,-6,-16	(29)
Turkey	50	32.0	HPV-33,HPV-18	(30)
India	21	9.5	HPV-16	(35)

*Not a random sampling: presence of coilocytes in the samples.

**Table 5:** Data of papillomaviruses (HPV) DNA detection in non-malignant neoplasms of mammary gland.

Country	Number of samples, n	HPV+samples,%	Predominant HPV type	Reference
Iran	150	0	–	(27)
Spain	186	26.3	HPV-16	(33)
India	10	30.0	HPV-16	(35)
MpaH Iran	11	0	–	(39)
USA	21	0	—	(43)
Australia	10	10.0	HPV-18	(50)
Thailand	350	2.9	HPV-16	(53)
Morocco	12	8.3	HPV-5 (non-oncogenic, genus β)	(55)
Qatarr	50	8.0	HPV-35	(58)

When HPV16 was detected in non-tumor breast tissue adjacent to breast cancer, if the tumor was HPV-positive, then 7 out of 11 studied normal tissue samples also tested positive for HPV; if the breast cancer was HPV-negative, no viral DNA was detected in any of the 11 analyzed adjacent tissue samples (45). Human papillomavirus DNA has also been detected in some non-malignant breast epithelial samples with pathological changes (**Table 5**). In all studies, the HPV positivity rate in benign breast tumors did not exceed that in breast cancer, being 24.8%, 16.0%, 51.8%, 4.3%, 0%, 11.8%, 25.0%, 10.0%, and 63.9%, respectively (see **Table 1**). The HPV types detected in these samples usually matched those identified by the researchers in breast cancer.

Given the substantial heterogeneity among studies, we attempted to describe the data in a stratified manner. Roughly divided by geographic region, HPV positivity rates in some Asian studies (e.g., India, Iran, China, Thailand) fluctuated between 0% and about 64%, with a large study from India (35) reporting a high positivity rate of 63.9%, while studies from Iran (27) and Thailand (53) reported low rates of 0% and 4.3%, respectively. Studies from Europe and North America (e.g., Italy, Spain, USA, Greece) reported positivity rates ranging from about 15% to 52%. Studies from Australia (34, 46, 50, 60) also showed wide variations in positivity rates (from 5.9% to 78.6%). Analysis by main detection method (PCR primer type) indicates that studies using universal primers like GP5+/6+ or MY09/11 typically detect a broader spectrum of HPV types, but sensitivity may vary by laboratory conditions; whereas studies using type-specific primers (e.g., for HPV16/18 E6/E7) or real-time quantitative PCR may be more sensitive for specific types but may miss others. This difference in detection technology is one of the key technical reasons for the variation in positivity rates.

## State of the human papillomavirus genome in breast cancer

In cervical cancer, besides episomal human papillomavirus, viral forms integrated into the host cell genome have been identified (35, 38). To verify this, researchers examine the disruption of the E2 open reading frame in the viral genome. The rationale is that during transformation of cervical epithelium, the viral genome integrates into host chromosomes at a specific stage of cervical intraepithelial neoplasia (CIN) progression; the breakpoint at the 3’ end of the viral genome may vary among tumors but often lies within the E1-E2 region, rendering the E2 reading frame undetectable by PCR; transcription of the viral oncogenes E6 and E7 is enhanced, and the presence of integrated HPV genomes may be a prognostic indicator for progression of specific intraepithelial neoplasia to cancer, as many CIN lesions regress spontaneously (62–65).

In the vast majority of cases, the human papillomavirus genome exists in an integrated state in breast tissue. S. Islam and colleagues, by assessing the E2/E6 copy number ratio, found that among 120 HPV-positive breast tissue samples, 87.5% contained integrated HPV, 8.3% were in a mixed state (i.e., both integrated and episomal HPV coexisted), and 4.2% contained only episomal HPV (35). N. Khodabandehlou and colleagues measured the proportion of samples expressing E6 that also expressed E2. Among 35 HPV-positive samples, 86% were determined to contain only integrated HPV genomes, and the remaining 14% were mixed (38). The biological significance of HPV genome integration into the host cell genome remains debated: specifically, whether integration is a necessary condition for malignant cell transformation or merely an accompanying phenomenon. In cervical epithelium, integration sites are numerous and non-randomly distributed—they tend to cluster in gene-rich regions, chromosomal fragile sites, enhancers, and transcriptionally active regions (66). Clearly, integration of the HPV genome into the cellular genome affects the expression of both viral and host genes and increases host genome instability. HPV genome integration into host chromosomes is seen in various cancer types, and research has confirmed the role of these viruses in the carcinogenesis of those cancers (67–69).

## Viral load in papillomavirus-positive breast cancer samples

Multiple research teams have quantified the number of human papillomavirus genomes (i.e., viral load) in breast tissues testing positive for the virus using real-time polymerase chain reaction (qPCR) (35, 45, 48). Viral load ranged from 5.4 to 6.5 HPV genome copies per 10,000 cells (35, 45). For rare cases of HPV-positive breast metaplastic sarcomatoid carcinoma, viral load was relatively high, reaching 204.03 copies per 10,000 cells in metaplastic sarcomatoid carcinoma with chondroid differentiation and 10,152.11 copies per 10,000 cells in metaplastic sarcomatoid carcinoma with squamous differentiation. Considering the overall differentiation status, the average viral load was 2,089.2 copies per 10,000 cells (48).

In cervical cancer, this indicator is 130,480 human papillomavirus genome copies per 10,000 cells (45). In the HPV16-positive cell line SiHa derived from cervical cancer tissue, the viral load is 39,850 HPV copies per 10,000 cells (48). In both cases, there is at least one viral genome copy per cell, sufficient to maintain the malignant phenotype. Notably, the viral load in HPV-positive breast cancer is significantly lower than one copy per cell. Given that breast cancer is considered a monoclonal tumor and the HPV genome integrates into the host genome only once, it should not disappear during tumor cell replication; therefore, at least one HPV copy per tumor cell would be expected. Researchers’ interpretations of the low HPV load in breast cancer diverge: some scholars believe this indicates HPV does not play a primary role in breast cancer development (45), while others suggest it indicates a different role for HPV in breast carcinogenesis compared to cervical cancer carcinogenesis. For example, they might invade already malignantly transformed breast tissue during an early pre-clinical stage of the malignant transformation process and influence its progression (35, 48).

The relatively low level of human papillomavirus load in breast cancer-positive samples raises questions about whether carcinogenic HPV participates in the tumorigenic process. Related studies indicate that the detection of the DNA cytidine deaminase APOBEC3B in breast cancer and the influence of carcinogenic HPV on the expression of this enzyme have largely addressed such doubts (70, 71).

Breast carcinogenesis relies on somatic mutations, with C→T transition mutations playing a dominant role. According to the findings of M.B. Burns and colleagues (70), a potential source of these mutations in breast cancer is the cytidine deaminase APOBEC3B DNA. In 38 breast cancer cell lines and primary breast cancer cells, A3B messenger RNA content was at least three times higher in 28 cases compared to controls (breast tissue from reduction mammoplasty), and ten times higher in 12 cases. Tumors with higher A3B mRNA content had twice the number of mutations compared to those with lower content. In cytoplasmic extracts from breast cancer cell lines, endogenous A3B was the sole source of C→T editing activity. When A3B expression was induced to upregulate, abnormalities such as cell cycle disruption, cell death, DNA fragmentation, and increased C→T transitions were observed. Based on the results, the authors constructed a model suggesting that A3B-catalyzed deamination provides a continuous source of DNA damage for breast cancer cells, leading to selection for TP53 inactivation; this model explains why some tumors progress rapidly and exhibit heterogeneity (70).

K. Ohba and colleagues (71) investigated whether carcinogenic human papillomavirus could be an inducing factor in breast cancer development by triggering APOBEC3B overexpression, thus serving as the missing link between HPV and breast cancer progression. The researchers used normal mammary epithelial cells transfected with HPV18 and observed overexpression of APOBEC3B messenger RNA. Compared to the original mammary cells without the HPV18 genome, the presence of the viral genome in this experimental system led to increased expression levels. A3B expression increased by 3.5-fold. Simultaneously, the expression of other cytidine deaminases was suppressed, indicating that the enhancement of A3B expression was specific. In breast cancer cells transfected with human papillomavirus type 18, A3B overexpression was accompanied by increased genomic instability (demonstrated by DNA comet assay); this effect could be inhibited by small interfering RNA targeting HPV18 E6 and E7, as well as A3B (gene knockdown confirmed by reverse transcription quantitative PCR). After obtaining these results *in vitro*, the researchers investigated A3B expression levels in HPV18-positive and negative breast cancer samples. The trend observed *in vitro* was reflected in this case as well. Overall, the researchers interpreted these results as evidence supporting HPV involvement in the early stages of breast cancer development (71). Simultaneously, the observations of J.S. Lawson and colleagues are noteworthy. In immunohistochemical detection, they used an antibody against HPV18 E7. The researchers pre-screened a group of women who had a history of benign breast tumors at the time of breast cancer diagnosis. They compared E7 expression in tissue samples from each woman (including both the cancer sample and the previous benign tumor sample). In breast cancer patients, compared to the same patient’s prior benign breast tumor, expression of this viral oncoprotein was markedly weaker in some cases’ tumors, and in some patients’ breast cancer samples, the protein was even completely absent (60).

The aforementioned studies suggest that the role of carcinogenic human papillomavirus in breast cancer pathogenesis is fundamentally different from its role in cervical cancer pathogenesis: breast cancer development does not require the persistent presence and expression of the viral genome. In breast tissue, HPV plays a role in the early stage of the disease, and its induction of apolipoprotein B mRNA-editing enzyme catalytic polypeptide-like 3B (APOBEC3B) can lead to genomic instability, thereby inducing breast cancer development.

## Histological and clinical characteristics of papillomavirus-positive breast cancer

At the initial diagnosis stage, breast cancer patients whose tumor tissues contain carcinogenic human papillomavirus DNA are significantly younger than those with HPV-negative tumors. For example, according to the results of C. Kroupis and colleagues, whose study primarily detected HPV type 16, the average age of HPV-positive breast cancer patients was 38 years (range 35-51), while the average age of HPV-negative women was 53 years (range 44-63) (p = 0.001) (44). J.S. Lawson and colleagues analyzed an Australian cohort of breast cancer patients previously diagnosed with cervical erosion (34). Compared to the average age of breast cancer onset in the Australian population, these patients developed breast cancer at a significantly younger age, 51 vs. 60 years; in this non-random sample, approximately every other woman had breast cancer, 78.6% of breast cancer samples were HPV-positive, and HPV type 18 predominated in both cervical epithelium and breast cancer.

According to the immunohistochemical detection results of C. Kroupis and colleagues, human papillomavirus-positive tumors exhibited relatively weaker estrogen receptor expression (p < 0.009) and stronger proliferative activity. This study found no correlation between progesterone receptor expression and HPV status (p = 0.92). Among HPV-positive breast cancer cases, poorly differentiated (grade III) cases constituted the majority, reaching 70.6%; whereas in HPV-negative cases, this proportion was 33.3%, a statistically significant difference (p = 0.005). Ductal carcinoma was the predominant histological type in both HPV-positive and negative breast cancer (44). However, some studies reported no correlation between HPV-positive breast cancer and tumor histological type or disease stage (72).

S. Islam and colleagues conducted a study using the Kaplan-Meier method, reporting that untreated high-risk HPV DNA-positive breast cancer patients had a worse prognosis compared to HPV-negative cases (p = 0.04) (35). The authors observed a similar trend in the cohort of treated patients as well.

Overall, existing data indicate that human papillomavirus DNA-positive breast cancers exhibit distinct histopathological and clinical features. Given the limited number of related studies, the existence of these characteristics clearly requires further validation.

## Association vs. causation: interpreting existing evidence with caution

**Figure 1:**
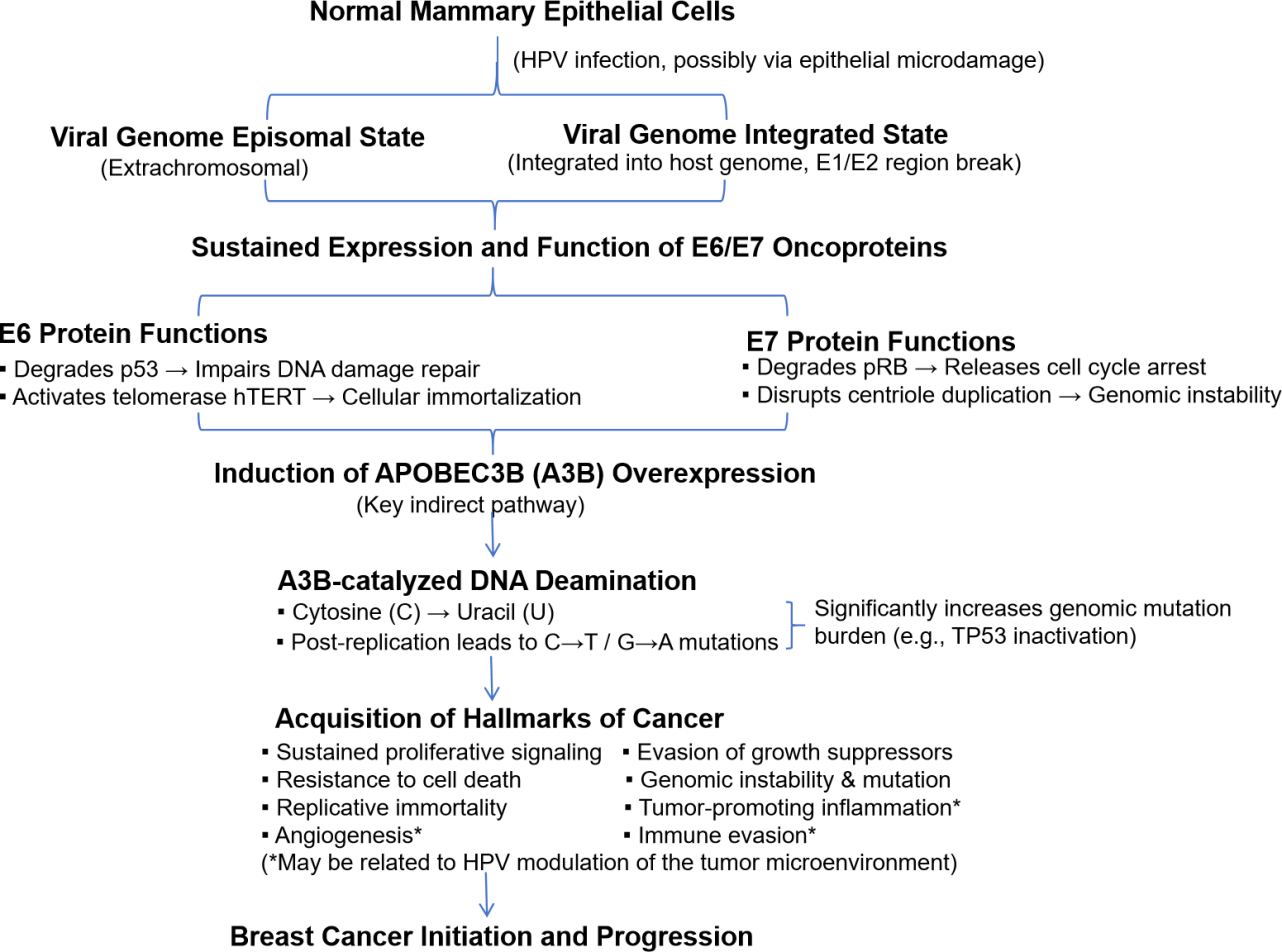
Schematic diagram of the potential mechanisms of HPV action in breast cancer development.

Although this review summarizes a large body of research finding HPV DNA present in a subset of breast cancer tissues, it is essential to carefully distinguish between association and causation. Firstly, HPV has also been detected in normal breast tissue (**Tables 3**–**5**) as well as in benign lesions, albeit usually at lower rates than in adjacent cancerous tissue, indicating that the mere presence of HPV is insufficient to cause cancer. Secondly, most current evidence comes from cross-sectional or case-control studies, designs that cannot establish temporal sequence, i.e., they cannot clarify whether HPV infection occurred before, after, or concurrently with carcinogenesis. Thirdly, even with evidence of viral integration and viral oncoprotein expression (e.g., E6/E7), the direct causal link between these events and the malignant phenotype in breast cancer has not been as rigorously established as it has in cervical cancer. For instance, the phenomenon of extremely low viral load suggests that HPV may not be the “initiating factor” driving all tumor cell clones, but more likely acts as a “promoter” or “modifier” during the early or late stages of tumor development, potentially through mechanisms such as inducing genomic instability (e.g., via APOBEC3B activation), i.e., the “oncomodulation” hypothesis (15, 71). This mode of action might explain why in some studies, viral oncoprotein expression in cancer tissue was even lower than in the same patient’s prior benign lesion (60). Therefore, while the association between HPV and breast cancer deserves attention, particularly its potential to induce gene mutations through mechanisms like APOBEC3B (see **Figure 1**) (70, 71), establishing it as a definitive cause of breast cancer requires more prospective cohort studies, mechanistic research, and evidence meeting Bradford Hill criteria (such as dose-response relationship, specificity, biological plausibility, etc.).

## Conclusion

This review, by integrating 34 global studies, provides comprehensive evidence for the association between carcinogenic human papillomavirus and breast cancer and proposes a groundbreaking mechanistic explanation: HPV may drive tumorigenesis via a unique “low viral load, high carcinogenic impact” mode by activating the host APOBEC3B enzyme to induce genomic instability. However, it must be emphasized that existing evidence primarily shows an association; establishing causation still requires further research. The presence of HPV in normal tissues, the substantial heterogeneity among studies, and the lack of prospective evidence are all factors that require cautious interpretation. If future research can more definitively establish an early role for HPV in breast carcinogenesis, this finding would provide a compelling theoretical basis for existing HPV vaccines to prevent a subset of breast cancers and potentially open new paradigms in cancer prevention.
